# Misinformation about COVID-19 among middle-aged and older migrants residing in Brazil and Portugal

**DOI:** 10.1590/1980-220X-REEUSP-2022-0401en

**Published:** 2023-08-14

**Authors:** Rodrigo Mota de Oliveira, Álvaro Francisco Lopes de Sousa, Anderson Reis de Sousa, Agostinho Antônio Cruz Araújo, Vinícius de Oliveira Muniz, Inês Fronteira, Isabel Amélia Costa Mendes

**Affiliations:** 1 Universidade de São Paulo, Escola de Enfermagem de Ribeirão Preto, Ribeirão Preto, SP, Brazil.; 2 Hospital Sírio-Libanês, Instituto de Ensino e Pesquisa, São Paulo, SP, Brazil.; 3 Universidade Federal da Bahia, Escola de Enfermagem, Salvador, BA, Brazil.; 4 Instituto Ensinar Brasil, Serra, ES, Brazil.; 5 Universidade NOVA de Lisboa, Escola Nacional de Saúde Pública, Lisboa, Portugal.

**Keywords:** Communication, COVID-19, Aged, Transients and Migrants, Public Health, Comunicación, COVID-19, Anciano, Migrantes, Salud Pública, Comunicação, COVID-19, Idoso, Migrantes, Saúde Pública

## Abstract

**Objective:**

The aim of this study was to assess the prevalence of COVID-19 misinformation among migrants aged 50 or older residing in Brazil and Portugal.

**Method:**

This was a cross-sectional analytical study conducted among migrants from Portuguese-speaking countries living in Brazil and Portugal, who were 50 years of age or older. The prevalence ratios (PR) were estimated using the Poisson regression model.

**Results:**

Out of the 304 participants included in the study, 188 (61.8%) agreed with at least one piece of misinformation. Factors such as having a religious affiliation (aPR: 1.24), higher educational attainment (aPR: 1.17), knowing someone who died from COVID-19 (aPR: 1.78), and having no intention to get vaccinated (aPR: 1.36) were associated with a higher likelihood of agreeing with COVID-19 misinformation.

**Conclusion:**

The findings suggest that access to misinformation was influenced by social, economic, and religious factors among elderly migrants with low digital literacy, thus contributing to the dissemination of false content within this population.

## RESUMO

**Objetivo:**

Avaliar os conteúdos de desinformação relacionados a COVID-19 entre migrantes com 50 ou mais anos de idade residentes no Brasil e em Portugal.

**Método:**

Estudo transversal e analítico, com migrantes de países falantes do português residentes no Brasil e em Portugal que possuíam 50 anos ou mais de idade. O modelo de regressão de Poisson foi utilizado para estimar as razões de prevalência (RP).

**Resultados:**

Dos 304 participantes deste estudo, 188 (61,8%) concordaram com ao menos um conteúdo de desinformação. Ter uma religião (aPR:1,24), maior escolaridade (aPR:1,17), conhecer alguém que morreu decorrente da COVID-19 (aPR:1,78) e não pretender se vacinar (aPR:1,36) influenciaram a concordância com a desinformação da COVID-19.

**Conclusão:**

O acesso às desinformações esteve relacionado com aspectos sociais, econômicos e religiosos dos envolvidos e contribuíram para que a população idosa migratória de baixo letramento digital, pulverizasse conteúdos falsos entre outras pessoas.

## RESUMEN

**Objetivo:**

Evaluar los contenidos de desinformación relacionados con COVID-19 entre migrantes de 50 años o más que residen en Brasil y Portugal.

**Método:**

Estudio transversal y analítico con migrantes de países de habla portuguesa que residen en Brasil y Portugal y tienen 50 años o más. Se utilizó el modelo de regresión de Poisson para estimar las razones de prevalencia (RP).

**Resultados:**

De los 304 participantes en este estudio, 188 (61,8%) estuvieron de acuerdo con al menos un contenido de desinformación. Tener una religión (aPR:1,24), mayor nivel educativo (aPR:1,17), conocer a alguien que murió a causa de COVID-19 (aPR:1,78) y no tener la intención de vacunarse (aPR:1,36) influyeron en la concordancia con la desinformación sobre COVID-19.

**Conclusión:**

El acceso a la desinformación estuvo relacionado con aspectos sociales, económicos y religiosos de los participantes y contribuyó a que la población migrante de edad avanzada y con bajo nivel educativo digital difundiera contenido falso entre otras personas.

## INTRODUCTION

Since the emergence of SARS-CoV-2 in Wuhan, China, there has been a deluge of information pertaining to the COVID-19 pandemic disseminated through online platforms. This surge in information mirrors the rapid and widespread transmission of the virus itself. Various forms of half-truths and misinformation, including conspiracy theories, false testimonies, and the endorsement of pseudoscientific remedies for COVID-19, quickly gained traction and spread virally, amplified by the prevalence of social media^(1,2)^. Consequently, this has significantly impacted the response to the pandemic by affected populations^(3)^.

In recent years, there has been a burgeoning body of research focusing on the nature of COVID-19-related misinformation^(4)^. However, there remains a dearth of studies that delve deeper into comprehending how individuals, with their distinct and collective characteristics, influence their response to a severe wave of misinformation^(5,6)^. Among the characteristics under scrutiny, the impact of age on misinformation remains a challenge, as it generates nuanced and sometimes contradictory insights. Research exploring the relationship between age and misinformation has intensified over the past three decades; nevertheless, a consensus on whether older adults or younger individuals are more susceptible to misinformation has yet to be reached^(7,8,9,10)^.

Meta-analytic studies examining the effects of aging and misinformation have revealed that older adults, particularly those aged over 65, exhibit a heightened vulnerability to misinformation compared to other demographic groups. Another review study suggests that older adults not only display diminished abilities to accurately recall the source of original information but also demonstrate greater confidence in false memories, rendering them more prone to misinformation. Similarly, a study utilizing Twitter found that middle-aged users (50 years old and above) accounted for 80% of the dissemination of fake news^(11)^.

However, there is a paucity of literature focused on understanding the consumption of COVID-19-related misinformation among elderly migrants, despite recognizing the cumulative vulnerabilities associated with this population. Migrants undergo disruptions in their life trajectory, leaving behind the sociocultural contexts to which they were previously accustomed, and which provided them with a sense of security and meaning^(12)^. Nevertheless, in the case of older migrants, various factors operating at macro, meso, and micro levels shape their vulnerabilities and coping mechanisms in the face of adversity. These factors encompass economic conditions in their countries of origin and destination, as well as individual factors such as migration experience, socioeconomic status, occupation, health condition, language proficiency, technological literacy, and health literacy^(12,13)^.

There is an increasing recognition that migrants are becoming a substantial segment of the elderly population in Europe and America. Between 2010 and 2015, there was a notable increase in the number of foreign-born residents aged 55 or older across Europe. Among the selected countries, Finland, Luxembourg, and Portugal exhibited a growth rate of over 50% in the older migrant population. Similarly, significant numbers and a consistent upward trend can be observed among foreign-born individuals in these countries within the age group of 45 to 54, referred to as the “future older migrants”^(14)^.

The aim of this study is to evaluate the content of COVID-19-related misinformation among migrants aged 50 or older residing in Brazil and Portugal.

## METHODS

### Study Design

This study employed an analytical design as part of a multicenter online project titled “Fact or Fake - Covid-19 Infection in Portuguese-Speaking Countries: Knowledge, Acceptability, and Impact of Fake News on Country Responses.”

### Population

The study included migrants born in one of the seven Portuguese-speaking countries (Brazil, Portugal, Angola, Cape Verde, Guinea-Bissau, Mozambique, São Tomé and Príncipe) who had been residing in Portugal or Brazil for a minimum of three months and were 50 years of age or older. Tourists and domestic migrants were excluded from participation. The snowball method was adapted for online recruitment^(15)^, where participants took on the responsibility of recruiting others from the same category through their social networks. To ensure sample variability and meet methodological requirements, we randomly selected 30 individuals from Portugal and Brazil based on a database of previous studies^(3,16)^. These individuals formed the initial “seeds” of our sample and were intentionally diversified according to key factors such as geographical location within each country’s regions, native or migrant origin, race/ethnicity (white and non-white), age group (50-60 years, 61 or older), and educational level (elementary/middle school, high school, and postgraduate). The first 30 participants who agreed to participate were provided with the survey link and instructed to invite and share the link with others in their social network or social circles who were similar to them. This was done using an official invitation text and hyperlink sharing. The total population of the study consisted of 6,843 individuals, out of which 1,214 were 50 years of age or older. Among them, 304 (25.1%) were migrants residing in Brazil or Portugal and had provided complete and accurate responses to all study questions, rendering them eligible for participation in this study.

The decision to include individuals aged 50 or older was based on recommendations from previous studies^(10,17)^ and the understanding that this age group is relatively homogeneous in experiencing a decline in cognitive function as they age. This decline impacts their cognitive abilities, abstract reasoning skills, and their proficiency in navigating digital technologies, thereby limiting their ability to discern between accurate and misinformation content^(10)^.

### Study Variables

The primary outcome of this study was agreement with at least one piece of misinformation content. To achieve this, responses of “agree” and “strongly agree” were grouped together as “agreement” (3). Secondary variables included participants’ social and demographic characteristics, familiarity with COVID-19 (based on testing and knowing individuals who died from the virus), consumption of COVID-19-related content (information sources), decision-making based on content, and willingness to vaccinate^(3,16)^.

### Data Collection

The research was conducted in two phases between June and August 2020:

1) Data mining was performed to identify the main topics related to COVID-19 misinformation discussed in Portuguese, as described in previous studies^(3,16)^.

For conceptual purposes, and in accordance with relevant literature, the news articles were grouped into two categories: Category 1 – Conspiracy theories regarding the origin, prevention, treatment, and cure of COVID-19/SARS-CoV-2; and Category 2 – Homemade and non-pharmacological methods for preventing contagion and treating SARS-CoV-2.

Category 2 – An online population survey was conducted among individuals originating from the seven Portuguese-speaking countries (Brazil, Portugal, Angola, Cape Verde, Guinea-Bissau, Mozambique, São Tomé and Príncipe) but residing in Brazil or Portugal for more than three months. The objective of this phase was to assess agreement with the content published on the studied social media platforms, as selected in Phase 1^(3,16)^.

A structured questionnaire was developed by the authors based on the literature^(18,19,20)^ and was available in two versions: Brazilian Portuguese and European Portuguese. The questionnaire consisted mostly of multiple-choice questions and encompassed topics such as social and demographic information (age, country of origin, nationality, religion, education, living conditions), behaviors adopted to cope with the COVID-19 pandemic (social distancing, protective measures for COVID-19, adherence to those measures), information-seeking and consumption of COVID-19-related news and information, as well as 21 specific questions regarding agreement with the origin of SARS-CoV-2, prevention, treatment, and cure of COVID-19. To minimize information bias, for every seven incorrect questions, one correct question was included for validation.

The questionnaire was evaluated and validated by a panel of 10 expert judges, with five from each country, through two Delphi rounds to achieve consensus. The comprehensive expert analysis encompassed relevance, coherence, construct validity of the questionnaire, as well as its cultural and linguistic properties. The online questionnaire was hosted on a dedicated website that allowed for quick data collection in Brazilian and European Portuguese and permitted only one response per Internet Protocol (IP) address, thus ensuring a single response per electronic device and avoiding selection biases^(3)^.

### Data Analysis and Treatment

Data were analyzed using Statistical Package for the Social Sciences (SPSS) version 24.0 (SPSS Inc., Chicago, IL, USA). Descriptive analysis included absolute and relative frequencies. Prevalence ratios were used to assess crude associations (bivariate analysis), and their statistical significance was tested using Pearson’s chi-square test and the Monte Carlo method, considering a minimum significance level of p ≤ 0.05.

Ninety-five percent confidence intervals (95% CI) were also established. Monte Carlo permutations were used to calculate p-values for independent variables with more than two categories of analysis to achieve better statistical data fit. All variables were assessed for multicollinearity using tolerance coefficients and variance inflation factors (VIF). Given the high frequency of the reference outcome (agreement with COVID-19 misinformation greater than 10%), traditional logistic regression analysis using odds ratios (OR) overestimates associations. Therefore, a Poisson regression model with robust variance estimation using a covariance matrix (generalized linear model) was chosen to estimate the prevalence ratio (PR), which is the most appropriate measure for cross-sectional studies. A logarithmic link function and 95% CI were also employed. Variable selection for the multivariate model was based on the results of bivariate analysis, considering statistical significance (p-value ≤ 0.05), theoretical relevance, or better fit conditions. The observed parameters for the best performance adopted the Akaike information criterion (AIC), log-likelihood, omnibus test, and effect tests (Type III) as references.

### Ethical Aspects

The study was conducted in accordance with the research ethics regulations of the two participating countries. In the Brazilian context, it was approved by the Research Ethics Committee (Comissão de Ética em Pesquisa - CONEP) under opinion 4,950,793 in the year 2020. The study adhered to the Declaration of Helsinki and relevant legislation in each country, including Resolution 466/12. All participants provided online informed consent by signing a consent form.

## RESULTS

Among the 304 participants included in this study, 210 individuals (69.1%) fell within the age range of 50 to 59 years. The majority of participants were in a relationship (235; 77.3%), resided in homes with four to seven rooms (195; 64.1%), and lived with up to three people (242; 79.6%). In terms of education, a significant majority had received more than nine years of schooling (185; 60.9%), and a notable proportion did not adhere to any religious practices (169; 55.6%).

A total of 188 participants (61.8%) expressed agreement with at least one piece of misinformation. Among the two categories analyzed, Category 01 misinformation pertaining to the use of infrared light from a digital thermometer on the forehead, which purportedly leads to brain damage, hormonal issues, and insomnia, garnered the highest agreement percentage of 26.7%. In Category 2, the misinformation suggesting that drinking potable water every 15 minutes can expel the novel Coronavirus and prevent its migration to the lungs received an agreement rate of 6.6% (see Table 1).

**Table 1.** Agreement with misinformation content, categorized, among middle-aged and older migrants. Misinformation Content – Ribeirão Preto, SP, Brazil, 2022.

	Brazil		Portugal		Total	
	n	%	n	%	n	%
**Category 1 – Conspiracy Theories about the Origin, Prevention, Treatment, and Cure of COVID-19/SARS-CoV-2**						
1. The use of infrared light from a digital thermometer on the forehead region should be avoided as it may lead to brain damage, hormonal problems, and insomnia.	36	27.1	45	26.3	81	26.7
2. Asymptomatic individuals diagnosed with SARS-CoV-2 are not capable of transmitting the virus to others.	15	11.3	23	32.5	38	12.5
3. SARS-CoV-2 was developed in a laboratory by Chinese scientists with the intention of using it as a biological weapon.	29	21.8	15	8.8	44	14.2
4. The pharmaceutical industry intentionally propagated the spread of SARS-CoV-2 for the purpose of population control.	5	3.8	10	5.8	15	4.9
5. SARS-CoV-2 has undergone genetic manipulation and exhibits a structure similar to that of the HIV virus.	18	13.5	6	3.5	24	7.9
6. SARS-CoV-2 cannot survive at temperatures higher than 26 degrees Celsius.	9	6.8	17	9.9	26	8.6
7. Social isolation may weaken the immune system and facilitate SARS-CoV-2 infection.	16	12	28	16.4	44	14.5
8. Wearing a facial protective mask for SARS-CoV-2 prevention can increase the concentration of the virus in the blood, making it denser and rendering the person more susceptible to thrombosis.	1	0.8	3	1.8	4	1.3
9. Excessive and regular use of facial protective masks for SARS-CoV-2 prevention can cause suffocation and should therefore be avoided.	2	1.5	13	7.6	15	4.9
10. Holding one’s breath for 10 seconds is not an accurate indicator of whether a person has COVID-19.	1	0.8	9	5.3	10	3.3
11. Daily use of hand sanitizer with alcohol can be toxic and extremely detrimental to health.	9	6.8	17	9.9	26	8.6
**Category 2 – Home Remedies and Non-pharmacological Methods for COVID-19 Contagion Prevention and Treatment**						
12. Fennel tea, warm water, or whiskey are purported to have an effect in combating or protecting individuals against the novel Coronavirus.	2	1.5	–	–	2	0.7
13. Avocado tea, hibiscus tea, perfume spray, and whiskey are substances that some believe can prevent infection.	2	1.5	–	–	2	0.7
14. A concoction of garlic with boiled water is claimed to kill the virus.	–	–	2	1.5	2	0.7
15. Vinegar is suggested to be more effective than alcohol in preventing contamination.	–	–	2	1.5	2	0.7
16. Autohemotherapy (injecting one’s own blood infected with SARS-CoV-2 to stimulate the immune system for protection) is claimed to be highly effective.	–	–	9	5.3	9	3.0
17. Consuming potable water every 15 minutes is believed to expel the novel Coronavirus, preventing its migration to the lungs.	4	3	16	4.7	20	6.6
18. Gargling with warm water, salt, and vinegar is proposed as a potential cure for the virus, as it supposedly remains in the throat for only 4 days.	–	–	7	4.1	7	2.3
19. The novel Coronavirus is purportedly eliminable from the body through water ingestion and gargling with warm water, saline, or acidic solutions, thereby impeding the progression of the infection.	1	0.8	7	4.1	8	2.6
20. Combining hand sanitizer with hair gel is suggested to slow down or eliminate the virus.	2	1.5	1	0.6	3	1.0
21. Using hand sanitizer is claimed to be more efficient than washing hands with water and soap.	3	2.3	9	5.3	12	3.9

Table 2 provides a detailed analysis of the agreement levels regarding misinformation content. The data shows that individuals residing in Portugal demonstrated a higher agreement rate (59.6%), as well as those within the age range of 50 to 59 years (69.1%) and those with more than nine years of education (82.4%). Furthermore, familiarity with COVID-19, such as personal experiences such as undergoing testing, knowing individuals who have had COVID-19, knowing someone who died from COVID-19, or being hospitalized due to COVID-19, had a significant impact on the agreement levels with misinformation content.

**Table 2.** Bivariate analysis of agreement with misinformation content among middle-aged and older migrants - Ribeirão Preto, SP, Brazil, 2022.

	Agreement with misinformation content						
	Yes		No		Total	
	n	%	n	%	n	%	p-value*
**Country of residence**							0,86
Brazil	76	40,4	57	49,1	133	43,8	
Portugal	112	59,6	59	50,9	171	56,2	
**Age range**							0,97
50–59	130	69,1	80	69,0	210	69,1	
60 and above	58	30,9	36	31,0	94	30,9	
**Number of rooms in the house**							<0,001
1–3	34	18,1	45	38,8	79	26	
4–7	137	72,9	58	50	195	64,1	
8 or more	17	9,0	13	11,2	30	9,9	
**Number of household members**							0,149
1–3	144	76,6	98	84,5	242	79,6	
4–5	39	20,7	14	12,1	53	17,4	
6–8	5	2,7	4	3,4	9	3,0	
**Educational level**							0,199
Less than 9 years	33	17,6	14	12,1	47	15,5	
More than 9 years	155	82,4	102	87,9	257	84,5	
**Do you have a religion?**							0,001
Yes	97	51,6	38	32,8	135	44,4	
No	91	48,4	78	67,2	169	55,6	
**Have you been tested for COVID-19?**							0,065
Yes	66	35,1	29	25,0	95	31,3	
No	122	64,9	87	75,0	209	68,8	
**Do you know someone who has had COVID-19?**							0,012
Yes	104	55,3	47	40,5	151	49,7	
No	84	44,7	69	59,5	153	50,3	
**Do you know someone who has died from COVID-19?**							0,491
Yes	18	9,6	14	12,1	32	10,5	
No	170	90,4	102	87,9	272	89,5	
**Have you been hospitalized due to COVID-19?**							0,114
Yes	4	2,1	0	0,0	4	1,3	
No	184	97,9	116	100	300	98,7	
**During the COVID-19 pandemic, have you made any decisions based on non-scientific sources of information?**							0,017
No	144	76,6	104	89,7	248	81,6	
Yes, at least once	39	20,7	11	9,5	50	16,4	
**Do you intend to get vaccinated for COVID-19?**							0,21
Yes	143	76,1	100	86,2	243	79,9	
No	45	23,9	16	13,8	61	20,1	

*p = statistical significance.

The multivariate analysis identified important factors that can influence agreement with COVID-19 misinformation among migrants. Having a religion increased the prevalence of agreement with COVID-19 misinformation by 24%, while higher levels of education increased it by 17%. The factors that most significantly increased the prevalence of agreement with COVID-19 misinformation were knowing someone who died from COVID-19 (78%) and having no intention to get vaccinated (36%) (Table 3).

**Table 3.** Multivariate analysis of factors associated with agreement with misinformation content among older migrant individuals – Ribeirão Preto, SP, Brazil, 2022.

Variable		aPR^‡^	95% CI^§^	p-value*
**Religious affiliation**				0,02
No	1	1		
Yes	1.22	1.24	1.13–1.54	
**Education level**				0,04
Less than 9 years of education	1	1		
More than 9 years of education	1.52	1.17	1.12–1.90	
**Knowing someone who has died from COVID-19**				0,02
No	1	1		
Yes	1.26	1.78	1.20–2.13	
**Willingness to get vaccinated against COVID-19**				0,01
Intends to get vaccinated	1	1		
Does not intend to get vaccinated	1.29	1.36	1.14–1.75	

Notes: ^†^PR = prevalence ratio; ^‡^aPR = adjusted prevalence ratio; ^§^95% CI = 95% confidence interval; *p = statistical significance.

## DISCUSSION

This study aimed to assess the consumption of false information about COVID-19 among elderly migrants in Brazil and Portugal. It is noteworthy that both the characteristics of the studied population, which consists of elderly migrants, and the context of the pandemic contribute to the consumption of false information on social media and the adoption of preventive and curative behaviors.

Migration is recognized as a social determinant that impacts health and well-being, particularly affecting socially vulnerable groups such as migrants. While social conditions generally shape the health outcomes of populations, additional factors like lack of information about health rights and low health literacy contribute to the vulnerabilities experienced by migrants. Consequently, addressing the needs of this population is a global health priority and should be incorporated into nursing actions and interventions^(21)^.

The migratory process becomes even more intricate when it involves the elderly population. According to the International Migration 2020 Highlights report, in 2020, 12% of the 281 million migrants worldwide were aged 65 or older^(22)^. This fact highlights how the migration of older adults impacts healthcare systems in various countries, as well as economic, social, and financial systems^(23)^.

Given the complex global health scenarios faced by migrant populations, the World Health Organization identifies this group as vulnerable and neglected, urging governments to take supportive actions and reorient health systems to ensure migrants’ access to available services in host countries. This guidance emphasizes health as a universal human right and stresses the need for inclusive universal health coverage that addresses the disproportionate and detrimental effects of the COVID-19 pandemic on migrant populations^(21)^.

When considering the consumption of COVID-19 information, it is crucial to acknowledge the fragility of access to quality and reliable information within the context of migration. Factors such as variability of information sources, cultural interpretation issues (including belief systems and language), language variations, low levels of health literacy, digital literacy, economic vulnerability, partisanship, political ideology, and the emergence of anti-vaccine channels or movements contribute to this fragility^(8,24,25,26,27)^.

Among the significant factors influencing agreement with COVID-19 misinformation among migrants, social variables (such as religion and education), familiarity with COVID-19 (such as knowing someone who died from the disease), and willingness to be vaccinated were highlighted. These findings underscore the importance of gaining qualitative insights into the migrant population in the context of their respective territories.

Religious practices frequently emerge as a recurring theme in the literature associated with misinformation. This phenomenon may stem from the influence of religious conceptions of the world, political views, notions of newsworthiness, and discourses of truth. In the era of “post-truth,” older individuals may be particularly susceptible to believing in relative or circumstantial situations that appeal to their emotions and personal convictions, such as discourses from religious leaders, rather than relying on objective facts when forming public opinion^(20)^. Furthermore, if older adults reside in religious institutions, the likelihood of misinformation may be even higher due to the absence of efforts to combat misinformation and a lack of research examining the susceptibility of the elderly population to false online information. Such research could involve examining digital platforms, providing education on device use, and offering relevant reading materials related to COVID-19^(28)^.

Another crucial aspect to consider is the level of education among the elderly population and its relation to misinformation, especially in the context of complex events like health crises. Incorrect consumption of false information poses a direct threat to public health and emphasizes the need for interventions with the elderly, including those with higher levels of education, who are also exposed to misinformation facilitated by easy access to media platforms such as digital social networks.

Our study findings indicate that knowing someone who died from COVID-19 increases the likelihood of agreeing with at least one erroneous piece of information. The process of grieving holds implications not only for the deceased but also for individuals, as it represents a moment of redefining their existence within society^(29)^. However, during the COVID-19 pandemic, this process was frequently hindered or even prevented due to government prevention and control measures. Studies conducted with individuals who lost someone to COVID-19 have demonstrated that the absence of funeral rituals leads to negative outcomes in the grieving process, including depression and anxiety, making the grieving process more painful and incomplete^(29,30)^.

Sociohistorical aspects that explain epidemic diseases like COVID-19 have proven valuable in understanding how populations have responded to the pandemic. By elucidating representative acts that demonstrate how people construct arguments and perceptions to deny the disease and its impacts on life and social organization, especially when it does not directly affect their immediate circles, we gain insights that remain distant from the collective imagination. This knowledge can aid nursing and healthcare teams in developing educational strategies, particularly regarding older individuals who believe in misinformation and are more likely to be hesitant about vaccination.

Promoting population vaccination and adopting a pro- vaccine stance, including expanding access to vaccination coverage for all populations, including migrant populations, is a position that nursing and healthcare teams should take to protect society, especially vulnerable groups such as the elderly, who have been disproportionately affected by COVID-19 in some countries^(8)^. It is crucial to promote health literacy among elderly migrants regarding vaccine information to ensure that myths, conspiracy theories, and denialist discourses do not impede their desire to be vaccinated against diseases, particularly those with high transmission and mortality rates^(8)^, as observed in Brazil^(16)^.

Lastly, nursing and healthcare professionals play a significant role in recognizing behavioral patterns, levels of health literacy, and knowledge deficiencies among the elderly population. This understanding can contribute to enhancing the quality of health education targeting this important demographic. However, it is important to acknowledge the limitations of this study. Our participants were from countries with varying levels of socioeconomic development and disparate pandemic response policies. Furthermore, our findings are limited to migrants with internet access, which introduces selection bias to the study population and may significantly influence our results. These limitations highlight the importance of considering these issues when conducting web-based research.

## CONCLUSIONS

The spread of false information regarding COVID-19 among elderly migrants from Portuguese-speaking countries residing in Brazil or Portugal is marked by a multitude of conspiracy theories, political and economic speculations, and baseless sanitary measures. These inaccurate claims are predominantly found in digital content that lacks scientific evidence and verification. The accessibility of such misinformation is linked to specific social, economic, and religious factors among the individuals involved, thereby exacerbating the dissemination of falsehoods within the digitally low-literate elderly migrant population. This prevailing circumstance presents significant challenges in addressing misinformation and other forms of disinformation throughout the duration of the COVID-19 pandemic.
